# Description length guided nonlinear unified Granger causality analysis

**DOI:** 10.1162/netn_a_00316

**Published:** 2023-10-01

**Authors:** Fei Li, Qiang Lin, Xiaohu Zhao, Zhenghui Hu

**Affiliations:** Key Laboratory of Quantum Precision Measurement, College of Science, Zhejiang University of Technology, Hangzhou, China; Department of Radiology, Shanghai Fifth People’s Hospital, Fudan University, Shanghai, China

**Keywords:** Nonlinear modeling, Description length, Unified Granger causality analysis, Functional MRI

## Abstract

Most Granger causality analysis (GCA) methods still remain a two-stage scheme guided by different mathematical theories; both can actually be viewed as the same generalized model selection issues. Adhering to Occam’s razor, we present a unified GCA (uGCA) based on the minimum description length principle. In this research, considering the common existence of nonlinearity in functional brain networks, we incorporated the nonlinear modeling procedure into the proposed uGCA method, in which an approximate representation of Taylor’s expansion was adopted. Through synthetic data experiments, we revealed that nonlinear uGCA was obviously superior to its linear representation and the conventional GCA. Meanwhile, the nonlinear characteristics of high-order terms and cross-terms would be successively drowned out as noise levels increased. Then, in real fMRI data involving mental arithmetic tasks, we further illustrated that these nonlinear characteristics in fMRI data may indeed be drowned out at a high noise level, and hence a linear causal analysis procedure may be sufficient. Next, involving autism spectrum disorder patients data, compared with conventional GCA, the network property of causal connections obtained by uGCA methods appeared to be more consistent with clinical symptoms.

## Author Summary

In previous studies on functional brain networks, we have proposed a linear unified Granger causality analysis (uGCA) method based on description length. Considering that functional brain image data is characterized by nonlinear nonstationary signals with complex long- and short-range correlations, we thus proposed a nonlinear uGCA method. Then, through simulated data experiments and logical self-consistent fMRI experiments, its causality identification performance was demonstrated to be superior to that of conventional GCA. At the same time, we further revealed the phenomenon that nonlinear high-order characteristics and nonlinear cross-characteristics were submerged successively at high noise levels, indicating that linear modeling procedures might be sufficient to cope with causality studies based on fMRI data.

## INTRODUCTION

Granger causality analysis (GCA), as a data-driven test, is one of the most established methods to identify causal connections of brain networks. Specifically, GCA requires statistical significance to determine whether the unrestricted model provides a better prediction than the restricted model (C. Granger & Newbold, 1974; C. W. J. Granger, 1969). In general, through model selection technique, its optimal model basically is selected by balancing fitting error term and penalized term, then it uses *F* statistics to evaluate significance predictability. Therefore, most of the extensions of GCA are based on improvements in model selection techniques, *F* test, and modeling procedure (Shojaie & Fox, 2022). For example, since *F* test only involves bivariate variance comparison, numerous efforts have been made to scale from a small network with several nodes to a large-scale complex network (D’Souza, Abidin, Leistritz, & Wismüller, 2017; Marinazzo, Pellicoro, & Stramaglia, 2008; Wang et al., 2020; Zou, Ladroue, Guo, & Feng, 2010). And, to differentiate the causal effects of positive from negative, an asymmetric causality test was proposed (Hatemi-J, 2012). Moreover, causal investigation of GCA has been also generalized in other function spaces, for example, Fourier space (Geweke, 1982; Ning & Rathi, 2017; Stokes & Purdon, 2017). In addition, considering asymptotic noise distribution data, several forms of extensions, which are especially suitable in task-related fMRI studies, have been developed (Hacker & Hatemi-J, 2006; Kaminski, 2007). These extensions are essential since the causal effects between neuronal populations have complicated statistical interference due to various sources of uncertainties. So far, these methods have provided lots of profound insights for functional brain studies.

Nevertheless, most of these extensions are still in a conventional two-stage scheme: (1) specify model time-lag through Bayesian information criterion or Akaike information criterion (BIC/AIC), and then (2) identify causal effect by *F* test (S. Li, Xiao, Zhou, & Cai, 2018; Marinazzo et al., 2008; Tank, Covert, Foti, Shojaie, & Fox, 2018; Wismüller, Dsouza, Vosoughi, & Abidin, 2021; Yang, Wang, & Wang, 2017). This conventional scheme of GCA will lead to inconsistency in mathematical theories, subjective selection in confidence level, and repeated comparison of nested models, which will bring some performance issues. Specifically, different mathematical theories (BIC/AIC technique and *F* test) have different selection benchmark, which would bring some inconsistency in determining the optimal model. And selection results by pairwise *F* statistics sometimes depend on initially selected model and search path heavily. Meanwhile, it is worth noting that selecting and using *F* statistics have become very careful in current scientific researches, and its statistical significance has also caused extensive discussion (Amrhein, Greenland, & McShane, 2019; Benjamin et al., 2018; Wasserstein & Lazar, 2016). Another problem brought about by *F* test needs to be compared with each other through an intermediate model, which will increase algorithm complexity, especially in large-scale networks. Then, we referred to all these conventional two-stage GCA methods as the conventional GCA; in the comparison of different methods, the conditional GCA was used to deal with multivariate time series. But for as a matter of convenience, we named the conventional conditional GCA as conventional GCA in the following.

Adhering to Occam’s razor, we proposed a novel unified GCA (uGCA) (Hu, Li, Wang, & Lin, 2021; Li, Wang, Lin, & Hu, 2020); it rises above conventional GCA, in which the two-stage scheme can actually be viewed as the same generalized model selection issue. Specifically, with help of minimum description length (MDL) principle, uGCA unifies these two generalized model selection issues into a description length (or code length) guided framework. This means that it can integrate all candidate models into a unified framework with the same selection benchmark. In other words, the uGCA method integrates the whole causal investigation procedure together and straightway returns to the most suitable descriptive model by the description length. Then, through synthetic data and real fMRI data, we have verified and illustrated the superiority of uGCA method over conventional GCA in our previous study (Li et al., 2020).

Further on, based on different coding schemes, we present three different uGCA forms, uGCA-TP, uGCA-MIX, and uGCA-NML (Hu et al., 2021), and their large-scale network extension and dynamic network extension also have been developed (Hu, Li, Cheng, & Lin, 2022; F. Li et al., 2023). In general, we found that uGCA methods can identify true connections with a high accuracy, while eliminate false connections to a large extent. Meanwhile, we illustrated that uGCA-NML is the most recommended form (Hu et al., 2021). On the contrary, conventional GCA would cause lots of false positives whatever its confidence level, and it cannot guarantee a stable performance in true connection identifications, either.

On the other hand, it is well known that the nonlinear coupling phenomena commonly exist in complex brain networks (Bullmore & Sporns, 2009; Friston, 2000). But conventional GCA is performed in the context of linear autoregressive models for stationary time series. Therefore, nonlinear extensions of GCA have been paid lots of effort (Y. Chen, Rangarajan, Feng, & Ding, 2004; Farokhzadi, Hossein-Zadeh, & Soltanian-Zadeh, 2018; Y. Li, Wei, Billings, & Liao, 2012; Marinazzo et al., 2008; Tank et al., 2018), and two of the main approaches are the neural network–based extensions (Shojaie & Fox, 2022; Talebi, Nasrabadi, Mohammad-Rezazadeh, & Coben, 2019) and kernel-based extensions (Z. T. Chen, Zhang, Chan, & Schölkopf, 2014; Guo, Zeng, Shi, Deng, & Zhao, 2020; Liao, Marinazzo, Pan, Gong, & Chen, 2009). These nonlinear extensions have provided remarkable insights into functional brain networks, but they are essentially a conventional two-stage scheme, even though it improvements modeling procedure. Moreover, the prior knowledge about real connection networks of brain regions (neuronal populations) is insufficient, so their substantive coupling patterns are often difficult to capture due to the lack of ground truth. This means it may be harder to model the nonlinear characteristics of functional brain networks.

Immediately, we considered that a descriptive modeling procedure can be adopted, that is, using an approximate prediction model to approach the ground truth, which does not need the prior knowledge of nonlinear coupling function. Specifically, inspired by the idea of Taylor’s expansion, we decomposed nonlinear characteristics into two terms: high-order nonlinear term and cross-nonlinear term. Hence, we incorporated this nonlinear modeling procedure into our proposed unified method, which will be more available for causal investigating on nonlinear coupling networks. More significantly, we consider that some nonlinear characteristics of acquired brain imaging data may be easily drowned in high-noise environments. Therefore, through synthetic data and real fMRI data, we hope to demonstrate the superiority of nonlinear uGCA method over its linear representation and conventional two-stage GCA, while further verifying and illustrating these submerged phenomena.

## UNIFIED GRANGER CAUSALITY ANALYSIS

Inspired by coding theory, we proposed a novel causal investigation method based on coding candidate models, which to describe data models succinctly. This proposal unifies the two-stage scheme of conventional GCA into a description length guided framework by a single mathematical theory (Li et al., 2020), which can avoid some inherent issues in conventional GCA, such as the inconsistency of applying several mathematical theories, the subjective selection of confidence levels, and pairwise comparison of nested models, and so forth. Associating with the MDL principle, it provides a generic solution for model selection issues as a mathematical theory (Bryant & Cordero-Brana, 2000; Grünwald, Myung, & Pitt, 2005; Hansen & Yu, 2001), which regards probability distribution as a descriptive standpoint to choose a model with the shortest coding length scheme.

### Description Length Guided Linear Causal Modeling

Firstly, given two variables, *X*_*N*_ and *Y*_*N*_, the linear presentations about *X*_*N*_

$$\left\{\begin{array}{l} X_{t}=A_{1}X_{t-i}+\epsilon_{1t}\\ X_{t}=A_{2}X_{t-j}+B_{2}Y_{t-k}+\epsilon_{2t} \end{array}\right.$$ (1)

where *ϵ*_1*t*_, *ϵ*_2*t*_ are noise terms. *A*_1_, *A*_2_, and *B*_2_ are coefficient matrices. *X*_*t*_ denotes predicted vector (*n* * 1) at time *t*, *X*_*t*−*j*_ is explanatory vector, a *n* * *m* state matrix of variable *X* with time-lag *i*(*i* = 1, 2, …, *m*), same as *Y*_*t*−*k*_. Distilling the sense of Granger causality, if the joint historical information of *Y* and *X* can provide a better prediction to *X* than only using the historical information of *X* itself, then *Y* has Granger-cause to *X*. Thus, causal effect under the uGCA can be defined by

$$F_{Y\to X}=L_{X}-L_{X+Y}$$ (2)

where *L*_*X*_ denotes the shortest description length of restricted model in Equation 1, and *L*_*X*+*Y*_ denotes the shortest length of unrestricted model after adding *Y*_*N*_. Causal effect from *Y* to *X* existed when *F*_*Y*→*X*_ > 0, or else there is no causal effect.

### Conditional Granger Causality Concept

In order to remove spurious connections caused by indirect causal effects between nodes (L. Barnett, A. B. Barrett, & Seth, 2018; L. Barnett & Seth, 2014; Y. Chen, Bressler, & Ding, 2006), GCA also provides a measure of conditional causal connection by introducing another variable *Z*_*N*_ into Equation 1:

$$\left\{\begin{array}{l} X_{t}=A_{3}X_{t-i}+C_{3}Z_{t-j}+\epsilon_{3t}\\ X_{t}=A_{4}X_{t-k}+C_{4}Z_{t-p}+B_{4}Y_{t-q}+\epsilon_{4t} \end{array}\right.$$ (3)

where *A*, *B*, *C* are coefficient matrices, the *ϵ*_*t*_ are noise terms. Then, for conventional conditional GCA used for comparisons, the causal effect from *Y* to *X*, conditional on *Z*, is defined as

$$F_{Y\to X|Z}=\ln\frac{\text{var}\left(\epsilon_{3t}\right)}{\text{var}\left(\epsilon_{4t}\right)}.$$ (4)

Accessing conditional GCA notion, the effect from *Y* to *X* can also be identified while controlling the influence from another mediate node *Z* to *X*, in uGCA method (Li et al., 2020). That is, if *F*_*Y*→*X*_ > 0 existed, causal effect from *Y* to *X* conditioned *Z* is defined

$$F_{Y\to X|Z}=L_{X+Z}-L_{X+Y+Z}.$$ (5)

Thus in uGCA method, all candidate models can be compared freely in the context of their description length. Different from the conventional conditional GCA, the causal effects between multiple variables are obtained through repeated comparisons of nested models; uGCA can unified all candidate models into the same model space, the description length guided framework, and to obtain the conditional causal effect directly. For example, if both *F*_*Y*→*X*_ > 0 and *F*_*Z*→*X*_ > 0 existed,

$$F_{Y,Z\to X}=\min\left(L_{X+Y},L_{X+Z}\right)-L_{X+Y+Z}.$$ (6)

If *F*_*Y*,*Z*→*X*_ > 0, it means that both *Y* and *Z* have direct influence on *X*. But if *F*_*Y*,*Z*→*X*_ < 0, there will be two cases. One is *F*_*Y*,*Z*→*X*_ = (*L*_*X*+*Y*_ − *L*_*X*+*Y*+*Z*_) < 0; it indicates only *Y* has direct influence on *X*. The other is *F*_*Y*,*Z*→*X*_ = (*L*_*X*+*Z*_ − *L*_*X*+*Y*+*Z*_) < 0; it indicates that *Z* impacts *X* directly (Li et al., 2020). In the unified model selection space of uGCA, multiple selected models can be directly compared by their description lengths, which can release the algorithm complexity. In this way, the proposed uGCA is more in line with Occam’s razor, or the principle of *parsimony*.

### Description Length Models in uGCA

In our previous studies, we have present three different uGCA forms based on different coding schemes (Hu et al., 2021), further illustrating that uGCA-NML method is the most recommended choice (Hu et al., 2021). Thus, the following are generalized model selection strategies by description length in uGCA-NML. With the MDL principle, the shortest length of parametric model (that is, the *L* in Equation 2) is carried out. Variable *υ*^*n*^ = {*υ*_1_, …, *υ*_*n*_} is given,

$$\upsilon_{t}=\beta_{1}\upsilon_{t-1}+\beta_{2}\upsilon_{t-2}+\ldots +\beta_{k}\upsilon_{t-k}+\epsilon_{t}$$ (7)

where *t* = 1, …, *m*, and *m* is more than *k* to keep the solution determined. In earlier two-part coding scheme, it divided the descriptive model into a fitting error term (*L*_1_) and a parameter literal coding term (*L*_2_) (J. Rissanen, 1978), and its generalized description length is given by

$$L_{\text{two}–\text{part}}=L_{1}\left(\upsilon^{n}|F\right)+L_{2}\left(F\right)$$ (8)

where *F* denotes a probability distribution for the data set *υ*^*n*^. The parameter vector in Equation 7 consists of *θ* = (*k*, *ξ*) and *ξ* = (*σ*_2_, *β*_1_, …, *β*_*k*_), where *σ*^2^ = *ξ*_0_ is the variance parameter of zero-mean Gaussian distribution for *ϵ*_*t*_. In order to describe *υ*^*n*^, turn to describe *ϵ*_*t*_, thus it arrives at

$$f\left(\epsilon_{t}|\upsilon_{t},\beta ,\tau \right)=\frac{1}{{\left(2\pi \tau \right)}^{m/2}}e^{-\left(1/2\tau \right)\sum_{t}\left(\upsilon_{t}-\sum_{k}\beta_{k}\upsilon_{t-k}\right)}.$$ (9)

And the description length of two-part coding scheme for describing variable *υ*^*n*^ is

$$L\left(\upsilon ,\theta \right)=\ln\left(f\left(\epsilon_{t}|\upsilon_{t},\beta ,\tau \right)\right)+\sum_{i=0}^{k}\ln\frac{|\xi_{i}|}{\delta }+\ln\left(k+1\right)$$ (10)

where *δ* is the precision, it’s optimal to choose 1/$\sqrt{n}$. Specially, $\frac{|\xi_{i}|}{\delta }$ < 1 should be ignored.

### uGCA-NML-Minimax Solution for Inherent Redundancy

To remove some inherent redundancy in earlier two-part coding scheme, a sharper description length with stochastic complexity and universal process, combining Fisher information, is derived for a class of parametric processes (J. J. Rissanen, 1996), the so-called normalized maximum-likelihood (NML) coding scheme. In short, it applies an exponentiated asymptotic complexity instead of the parameter space complexity (the second part in two-part coding scheme, *L*_2_),

$$L_{NML}=-\ln\;f\left(\upsilon^{n}|\hat{\theta }\left(\upsilon^{n}\right)\right)+\ln\left[{\left(\frac{n}{2\pi }\right)}^{k/2}\cdot \int \sqrt{\det I\left(\theta \right)}d\theta \right].$$ (11)

This form is motivated by the maximum likelihood estimate (MLE) that requires satisfying the Central Limit Theorem (Barron, Rissanen, & Yu, 1998; J. Rissanen, 2000). Then, the nonintegrability of MLE is a key issue to be solved. Firstly, Fisher information is given by

$$|I\left(\beta ,\tau \right)|=|S|/\left(2\tau^{k+2}\right),$$

and the integral of its square root dealt by Barron et al. (1998), J. Rissanen (2000), and J. J. Rissanen (1996) is

$$\int_{\beta S\beta \leq R}\int_{\tau_{0}}^{\infty }{\left|I\left(\beta ,\tau \right)\right|}^{1/2}d\tau d\beta ={\left(2|S|\right)}^{1/2}{\left(\frac{R}{\tau_{0}}\right)}^{k/2}\frac{C_{k}}{k}.$$ (12)

Where *C*_*k*_*R*^$\frac{k}{2}$^ = 2(*πR*)^$\frac{k}{2}$^/(*k*$\sqrt{|S|}$Γ($\frac{k}{2}$)) is the volume of a k-dimensional ball *B* = {*β*′*Sβ* ≤ *R*}. Lower bound *τ*_0_ is determined by precision of data written, $\hat{\tau }$_0_ = *RSS*/*m* and $\hat{R}$ = ($\hat{\beta }$′$V_{t-k}^{\prime }$*V*_*t*−*k*_$\hat{\beta }$)/*m* are given by MLE. Finally, the description length in uGCA-NML arrives at

$$L_{\text{uGCA}-\text{NML}}=m\;\ln\sqrt{2\pi \tau }+\frac{RSS}{2\tau }+\frac{k}{2}\ln\frac{m}{2}-\log\;\Gamma \left(\frac{k}{2}\right)+\frac{k}{2}\log\frac{\hat{R}}{\tau_{0}}-2\;\log\;k.$$ (13)

### Nonlinear Characteristic Modeling Procedure

As stated above, most of modeling procedures fail to capture the nonlinear characteristics of neural coupling, which means that underlying functional brain networks may not be well uncovered. Thus, linear representation in Equation 1 should be described in nonlinear coupling form,

$$\left\{\begin{array}{l} X_{t}=f_{1}\left(X_{t-i}\right)+\epsilon_{1t}\\ X_{t}=f_{2}\left(X_{t-j}\right)+g_{2}\left(Y_{t-j}\right)+h_{2}\left(X_{t-q}Y_{t-r}\right)+\epsilon_{2t} \end{array}\right.$$ (14)

where *f*, *g*, and *h* denote underlying nonlinear coupling functions, and *X*_*t*−*i*_*Y*_*t*−*j*_ is the instantaneous cross-term.

It is well known that the current prior knowledge of real functional brain network is far from insufficient. And, in fact, from a methodological perspective, adopting an accurate and careful description model of causal effect may be more essential and urgent than searching a ground truth model.

As a result, in this study, we used an approximate modeling procedure to characterize the neural response, *f*, *g*, and *h*. Specifically, with the help of the idea of Taylor’s approximation expansion, their nonlinear characteristics can be decomposed into superpositions of high-order terms and cross-terms. That is,

$$X_{t}=\left[\begin{array}{cccc} X_{t,i} & X_{t,i}^{2} & \ldots & X_{t,i}^{s}\\ X_{t,i+1} & X_{t,i+1}^{2} & \ldots & X_{t,i+1}^{s}\\ \ldots & \ldots & \ldots & \ldots \\ X_{t,i+n} & X_{t,i+n}^{2} & \ldots & X_{t,i+n}^{s} \end{array}\right],Y_{t}=\left[\begin{array}{cccc} Y_{t,j} & Y_{t,j}^{2} & \ldots & Y_{t,j}^{s}\\ Y_{t,j+1} & Y_{t,j+1}^{2} & \ldots & Y_{t,j+1}^{s}\\ \ldots & \ldots & \ldots & \ldots \\ Y_{t,j+n} & Y_{t,j+n}^{2} & \ldots & Y_{t,j+n}^{s} \end{array}\right]$$ (15)

$$Z_{t}=\left[\beta ;X_{t};Y_{t};X_{t}^{\left(1\right)}Y_{t}^{\left(1\right)}\right]$$ (16)

where *X*_*t*,*i*_ is descriptive vector (*x*_*t*−1_, *x*_*t*−2_, …, *x*_*t*−*i*_) at time *t*, the same as *Y*_*t*,*j*_. And *s* is the polynomial order, *i* and *j* denote their time-lag. ***X***_***t***_ and ***Y***_***t***_ are the nonlinear representation matrices; ***Z***_***t***_ is the joint nonlinear representation matrix, where ***β*** denotes model coefficient vector. *X*_*t*_^(1)^ and *Y*_*t*_^(1)^ are the first column of ***Y***_***t***_ and ***Y***_***t***_, respectively. Actually, this nonlinear representation can be concluded as follow:

$$Z_{t}=\text{linearity}\;\text{term}+\text{high}‐\text{order}\;\text{term}+\text{cross}\;\text{term}$$ (17)

In this representation, we applied an approximate representation of Taylor’s expansion. The measured signal was decomposed into linear term and nonlinear terms, in which the nonlinear term was approximated by the high-order polynomial term and the cross-term. By the way, both conventional GCA and uGCA use the same nonlinear modeling procedure. In contrast to the linear modeling procedure, the nonlinear modeling procedure is essentially establishing a mixture descriptive model, in which it integrates nonlinear high-order terms and nonlinear cross-terms into a linear regression model.

In general, in the course of practical application, we used the least square method (LSQ) to estimate the model parameter of candidate models for both conventional GCA and uGCA methods. And different time delay orders in the model will get a different estimated model parameter. Then, for conventional GCA, it evaluates models with different estimation parameters according to BIC/AIC to obtain the optimal model, and then determines whether there is a causal relationship between variables through statistical inference. As for uGCA method, it is based on MDL principle, that is, by selecting the shortest description length among candidate models with different estimated parameters (e.g., in Equation 1) to determine the optimal models, and then by comparing the description length of selected optimal restricted model (in Equation 1) and the description length of selected optimal unrestricted model (in Equation 1) to establish the causal relationship. Therefore, their model estimations are consistent for both conventional GCA and uGCA (Barnett et al., 2018). Furthermore, in uGCA method, its model selection process and causal connection model construction were also under the same mathematical theory, which will obtain more consistent causal connection results.

## EXPERIMENTS AND RESULTS

### Synthetic Data Experiments

Nonlinear characteristics of high-order terms and cross terms may be easily drowned out by background noise. Therefore, through the synthetic data experiments, we hoped to demonstrated the priority of uGCA methods to conventional GCA within the nonlinear modeling procedure, and then attempted to illustrate that high-order terms and cross-terms in nonlinear systems were indeed drowned out successively with the increase of noise level.

#### 3-node network.

To ensure that the linear terms are the same and will not be affected by the added random noise term, we synthesize nonlinear data containing higher order terms and cross-terms, respectively, given by

$$\left\{\begin{array}{l} X_{1,i}=0.35X_{1,i-1}+0.34X_{2,i-1}+\epsilon_{1}\\ X_{1,i}^{h}=X_{1,i}-0.14X_{1,i-1}^{2}-0.14X_{2,i-1}^{2}\\ X_{1,i}^{c}=X_{1,i}-0.14X_{1,i-1}X_{2,i-1}\\ X_{2,i}=0.39X_{2,i-1}-0.36X_{1,i-1}+\epsilon_{2}\\ X_{2,i}^{h}=X_{2,i}-0.24X_{1,i-1}^{2}+0.24X_{2,i-1}^{2}\\ X_{2,i}^{c}=X_{2,i}-0.24X_{1,i-1}X_{2,i-1}\\ X_{3,i}=-0.37X_{3,i-1}+0.34X_{1,i-1}+0.35X_{2,i-1}+\epsilon_{3}\\ X_{3,i}^{h}=X_{3,i}+0.22X_{3,i-1}^{2}-0.21X_{1,i-1}^{2}-0.20X_{2,i-1}^{2}\\ X_{3,i}^{c}=X_{3,i}+0.21X_{1,i-1}X_{3,i-1}-0.20X_{2,i-1}X_{3,i-1} \end{array}\right.$$ (18)

where *X*^*h*^ represents variable *X* after adding high-order terms, and *X*^*c*^ is after adding cross-terms, *ϵ*_*i*_ is the noise terms. To ensure the effectiveness, synthetic data performed stationarity analysis and passed before being allowed to be further used.

**Figure 1.**
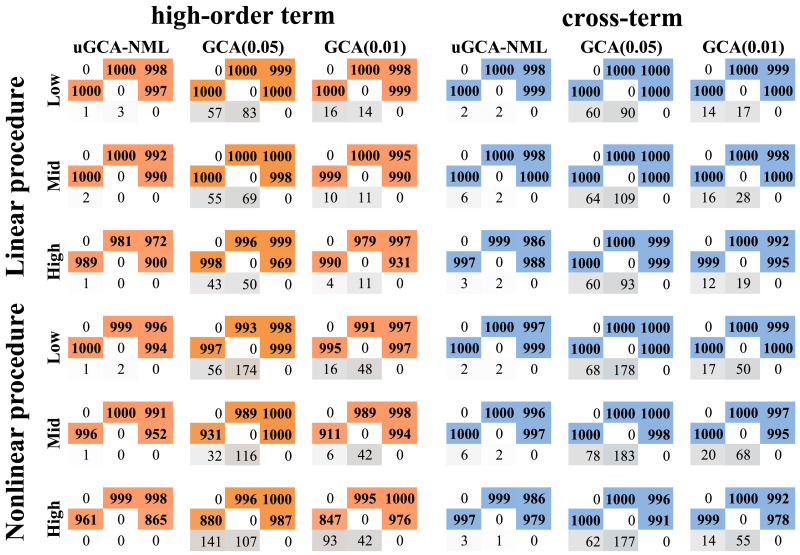
The obtained causal connections. Data length was 300, simulation number was 1,000. ‘Low’ denoted a low noise level (variance = 0.2), the noise variance in ‘Mid’ (‘high’) was 0.6 (1.2).

Firstly, for nonlinear data containing high-order terms, the results were shown in the left panel of Figure 1. Specifically, in terms of identifying true connections, the accuracy of linear uGCA-NML decreased significantly with the increase of noise level, especially in 2 → 3 and 1 → 3. Then, nonlinear uGCA-NML guaranteed a stable accuracy performance on the identification of 1 → 2 and 1 → 3 connections, but their accuracies in 2 → 1 and 2 → 3 had some descents. For the linear conventional GCA, their true prositive rates (TPRs) showed a descent of about 3% (a 7% descent in the high confidence level). After using nonlinear modeling, all accuracies of its four true connections had some descents, especially in 2 → 1. On the other hand, for these false connections, whether using nonlinear or linear modeling, uGCA-NML achieved more stable and accurate identification even at high noise levels. But for conventional GCA, its true negative rates (TNRs) were affected by noise terms to some extent, especially after using nonlinear modeling. Even if the confidence level was changed, the false connections identification performance of conventional GCA did not improve. Thus, as the noise level increased, the performance of all methods in identifying true connections decreased to some extent, whether using linear or nonlinear modeling. In other words, as the noise level increased, their high-order nonlinear characteristics would gradually be drowned out.

Then, for nonlinear data containing cross terms, the right panel of Figure 1 showed the cumulative result of all connection edges. Specifically, uGCA-NML behaved with superior identification performance in true connection whether using the linear or nonlinear modeling, and its accuracies were always maintained above 98%. Although, the accuracy of GCA-NML had a 2% decrease in 2 → 3 with the increase in noise level. Meanwhile, whether using linear or nonlinear modeling, uGCA-NML eliminated false connections to a large extent. On the other hand, conventional GCA had a good performance in identifying true connections, especially after using linear modeling. However, with the increase in noise level, its identification accuracy of some true connections had slight decreases, while the accuracy of false connections also fluctuated to some extent. With a high confidence level of the *F* test, conventional GCA eliminated false connections to a certain extent, but there would be more misjudgments in true connections. Therefore, with the increase in noise level, nonlinear characteristics with cross-terms will be also drowned to a certain extent.

**Figure 2.**
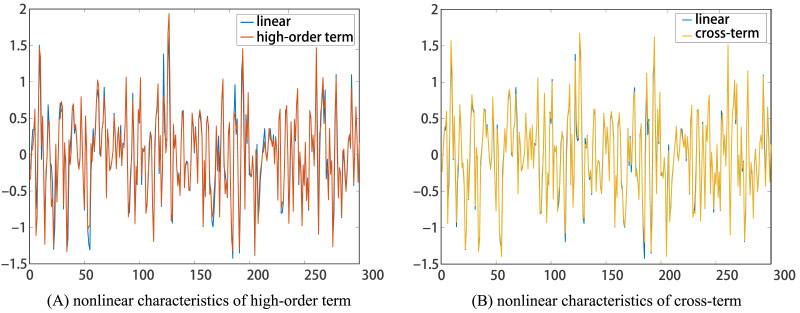
The comparison between linear data and nonlinear data with the high-order term (cross-term).

Therefore, as the noise level increased, more false negatives were made. And the high-order terms were significantly more affected by the increase in noise level than the cross-terms. Therefore, we considered that the nonlinear characteristics of high-order terms and cross-terms will be drowned out successively with the increase in noise level. And actually, these phenomena can be inferred in the time series diagram between linear data and nonlinear data, because they overlapped to a large extent, as seen in Figure 2. However, previous studies (Hu et al., 2021; Li et al., 2020) showed that identifying linear coupling would hold a stable level at the above noise ranges. In view of the fact that these obtained false negatives actually contained linear coupling characteristics, we considered that the *disorder* of nonlinear characteristics due to the increasing noise level may further cause the *loss* of linear characteristics. On the other hand, we found that uGCA-NML can ensure stable and superior performance in all identified connections, regardless of using linear or nonlinear modeling, while conventional GCA only obtains good identification in true connections.

#### 6-node network.

**Figure 3.**
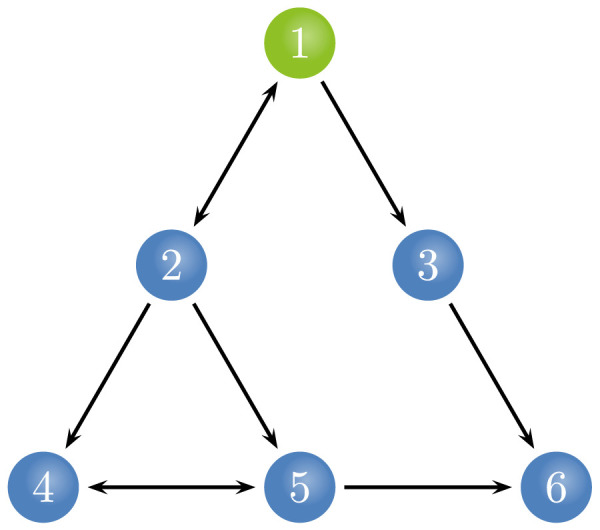
The relationships of simulation data sets in the six-node network.

The above results seemed to indicate that these methods have similar performance in causal connection identification through the linear or nonlinear modeling. Thus, a six-node network was further synthesized as follow, as shown in Figure 3,

$$\left\{\begin{array}{l} Y_{1,i}=0.85Y_{1,i-1}e^{-Y_{1,i-1}^{2}}-0.34Y_{1,i-2}+0.35Y_{2,i-2}+\epsilon_{1}\\ Y_{2,i}=0.96Y_{2,i-1}e^{-Y_{2,i-1}^{2}}-0.34Y_{2,i-2}+0.86Y_{1,i-1}+\epsilon_{2}\\ Y_{3,i}=0.82Y_{3,i-1}-0.36Y_{3,i-2}+0.81Y_{1,i-1}-0.32Y_{1,i-2}+\epsilon_{3}\\ Y_{4,i}=0.88Y_{4,i-1}e^{-Y_{4,i-1}^{2}}+0.32Y_{4,i-2}+0.89\text{tanh}(Y_{2,i-1})-0.84\text{tanh}(Y_{5,i-1})+\epsilon_{4}\\ Y_{5,i}=0.82Y_{5,i-1}e^{-Y_{5,i-1}^{2}}-0.59Y_{5,i-2}-0.48Y_{2,i-1}Y_{4,i-1}+0.43Y_{2,i-1}Y_{5,i-1}+0.45Y_{4,i-1}Y_{5,i-1}+\epsilon_{5}\\ Y_{6,i}=0.85Y_{6,i-1}e^{-Y_{6,i-1}^{2}}+0.26Y_{6,i-2}+0.82Y_{3,i-1}-0.55Y_{5,i-1}+0.22Y_{3,i-1}Y_{5,i-1}+\epsilon_{6} \end{array}\right.$$

**Figure 4.**
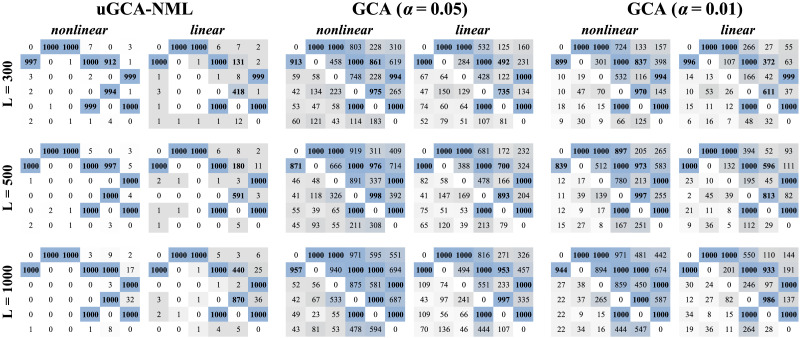
The obtained causal connections. The L denotes data length, its noise variance variance = 0.4.

Firstly, data length varied from 300 to 1,000, as shown in Figure 4, we found that both the uGCA and conventional GCA were affected by changes in data length, regardless of whether they used linear or nonlinear modeling procedure.

When data length increased, nonlinear conventional GCA obtained more true connections, but the misjudgment of some false connections increased, as same as using linear modeling. In general, when data length ranged from 300 to 1,000, linear conventional GCA cannot guarantee high accuracy in identifying true connections, especially 2 → 5, 4 → 5, and its false connections misjudgment was also relatively high. After using the nonlinear modeling, the identification performance of some true connections was improved, but lots of false negatives in 2 → 1, 2 → 5 still existed. And for false connections, there were also more very obvious misjudgments. More importantly, its identification performance cannot be improved even if its confidence level was changed. That is, when increasing the confidence level, conventional GCA (*α* = 0.01) eliminated false connections to some extents, but led to more false negative in identifying true connections, as seen in Figure 4.

But for nonlinear uGCA-NML, it obtained 100% accuracy of true connections when data length was 1,000, and the identification accuracy was also close to 100% as data length was 500. For most false connections, nonlinear uGCA-NML had stable and superior performance. But in some false connections, long data length led to some increases in false positives, especially in 2 → 6, 4 → 6. In general, it can be found that nonlinear uGCA-NML had a high identification accuracy of true connections, and it also suppressed false positives of false connections at a low level. As for linear uGCA-NML, their false positives were suppressed at a lower level than when using nonlinear modeling. However, for true connections 2 → 5, 4 → 5, linear uGCA-NML apparently failed to identify them well. Even when data length was 1,000, these two identification accuracies were significantly lower than that of other connected edges, especially for 2 → 5, its accuracy was always lower than 60%.

As shown in Table 1, with data length increased, uGCA-NML obtained a higher accuracy than conventional GCA, while their nonlinear modeling procedures were significantly better than linear procedures. In addition, when data length increased to 1,000, using nonlinear modeling resulted in a little increase in the misjudgment of some false connections, which led to a decrease in the ground truth rate. Even so, nonlinear uGCA-NML also obtained a much higher ground truth rate than that using linear modeling procedure. In general, nonlinear uGCA-NML can ensure high identification accuracy, and the possibility of obtaining a ground truth network is also significantly higher. In contrast, regardless of its confidence level, conventional GCA has a low probability of obtaining ground truth network whether using the nonlinear or linear modeling, and its ground truth rate is basically less than 10%.

**Table 1.** Comparison between uGCA methods and conventional GCA under different data length

Data length	Index	uGCA-NML		GCA(*α* = 0.05)		GCA(*α* = 0.01)	
		nonlinear	linear	nonlinear	linear	nonlinear	linear
L = 300	TPR	98.90%	83.87%	97.14%	91.41%	96.67%	88.64%
	TNR	99.86%	99.77%	76.89%	85.52%	85.95%	95.11%
	**Ground truth rate**	**88.0%**	**3.6%**	**0.3%**	**1.3%**	**2.1%**	**7.1%**
L = 500	TPR	99.97%	86.34%	98.28%	95.48%	97.88%	93.43%
	TNR	99.87%	99.79%	71.00%	81.73%	78.89%	93.08%
	**Ground truth rate**	**97.0%**	**7.6%**	**0.0%**	**1.1%**	**0.3%**	**11.4%**
L = 1000	TPR	100.00%	92.33%	99.52%	99.44%	99.38%	99.10%
	TNR	99.64%	99.54%	61.81%	75.80%	67.35%	89.21%
	**Ground truth rate**	**92.5%**	**32.8%**	**0.0%**	**0.5%**	**0.0%**	**9.4%**

*Note*. The L was data length. The ground-truth rate denoted the total numbers of the obtained individual connection network which was same as the ground truth network, divided by the sample number (1,000).

By changing noise levels, their accuracies were further directly compared when using nonlinear modeling and linear modeling, respectively, as shown in Table 2. Obviously, nonlinear uGCA-NML was basically not affected by noise terms, and it had a more stable and accurate performance of causal connection identification, especially for ground truth rate. In contrast, linear uGCA-NML cannot guarantee high identification accuracy, especially in TPR and ground truth rate. On the other hand, conventional GCA performed poorly whether using nonlinear or linear modeling, especially for the ground truth rate. As with changing the data length, its TPR had some decreases while the TNR and ground truth rate were improved when its confidence level was increased, although its ground truth remained at a low level. In general, nonlinear uGCA-NML was little affected by noise term, and can identify true connections with high accuracy while eliminating most false connection to a large extent.

**Table 2.** Comparison between uGCA methods and conventional GCA under different noise level

Data length	Index	uGCA-NML		GCA(*α* = 0.01)		GCA(*α* = 0.01)	
		nonlinear	linear	nonlinear	linear	nonlinear	linear
Low	TPR	99.99%	85.91%	99.40%	97.24%	99.16%	94.60%
	TNR	99.82%	99.77%	65.93%	75.22%	71.60%	89.38%
	**Ground truth rate**	**96.3%**	**9.4%**	**0.0%**	**0.3%**	**0.0%**	**6.9%**
Moderate	TPR	99.97%	86.34%	98.28%	95.48%	97.88%	93.43%
	TNR	99.87%	99.79%	71.00%	81.73%	78.89%	93.08%
	**Ground truth rate**	**97.0%**	**7.6%**	**0.0%**	**1.1%**	**0.3%**	**11.4%**
High	TPR	99.94%	85.82%	99.12%	94.34%	98.86%	92.61%
	TNR	99.78%	99.70%	69.23%	81.41%	75.64%	92.40%
	**Ground truth rate**	**95.3%**	**5.5%**	**0.0%**	**1.5%**	**0.0%**	**10.4%**

*Note*. ‘Low’ denoted a low noise level (variance = 0.2), the noise variance in ‘Moderate’ (‘High’) was 0.4 (0.6).

### fMRI Data

#### Mental arithmetic experiment.

In this experiment, we let nine subjects (five female, 24 ± 1.5 years old) perform simple one-digit (consisting of 1–10) serial addition (SSA) and complex two-digit (consisting of 1–5) serial addition (CSA) by visual stimulus and simultaneously measured their brain activities with fMRI. Immediately following, each subject was asked to perform the same serial addition arithmetic tasks by an auditory stimulus. Written informed consent was obtained from all participants. This study was approved by the local ethics committee of Zhejiang University of Technology.

It is well known that the obtained fMRI data are bound to be accompanied by highly nonlinear characteristics and often have large background noise. Although some studies have revealed that some noise of fMRI data may be potential neural activity signals of the brain (Bright & Murphy, 2015; Faisal, Selen, & Wolpert, 2008; Uddin, 2020), most of the noise is still objective. Especially for the inherent background imaging noise in fMRI, it is more likely to exist. Therefore, the nonlinear characteristics of coupled systems are likely to be drowned out in high noise level. Next, nonlinear uGCA-NML and nonlinear conventional GCA will be used to further analyze causal connection of mental arithmetic networks and verify whether nonlinear characteristics in fMRI data will be drowned out.

**Figure 5.**
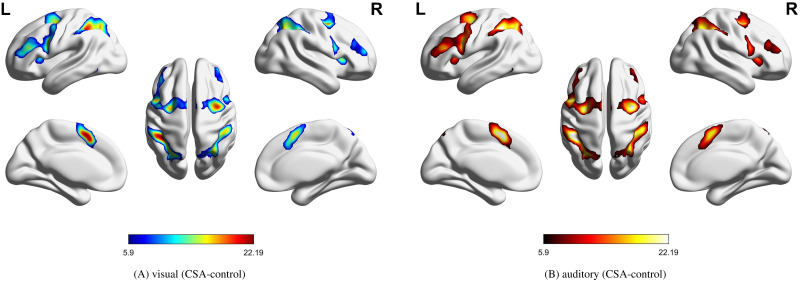
Mental arithmetic of CSA control state under the two stimuli. The activation regions were processed by SPM12; the control state meant that the sample was in rest state. (A) CSA control state under visual stimulus. (B) CSA control state under auditory stimulus (*p* < 0.0001, uncorrected).

Firstly, from a logical self-consistent view, we considered that mental arithmetic tasks would activate the same functional brain networks, whether under visual or auditory stimulus. Apparently, as seen in Figure 5, the activation maps under different stimuli overlapped to a large extent, which validated the reliability of the above experimental design. Thus, the effectiveness of these methods can be verified by measuring the similarity of their mental arithmetic networks under different stimuli.

**Figure 6.**
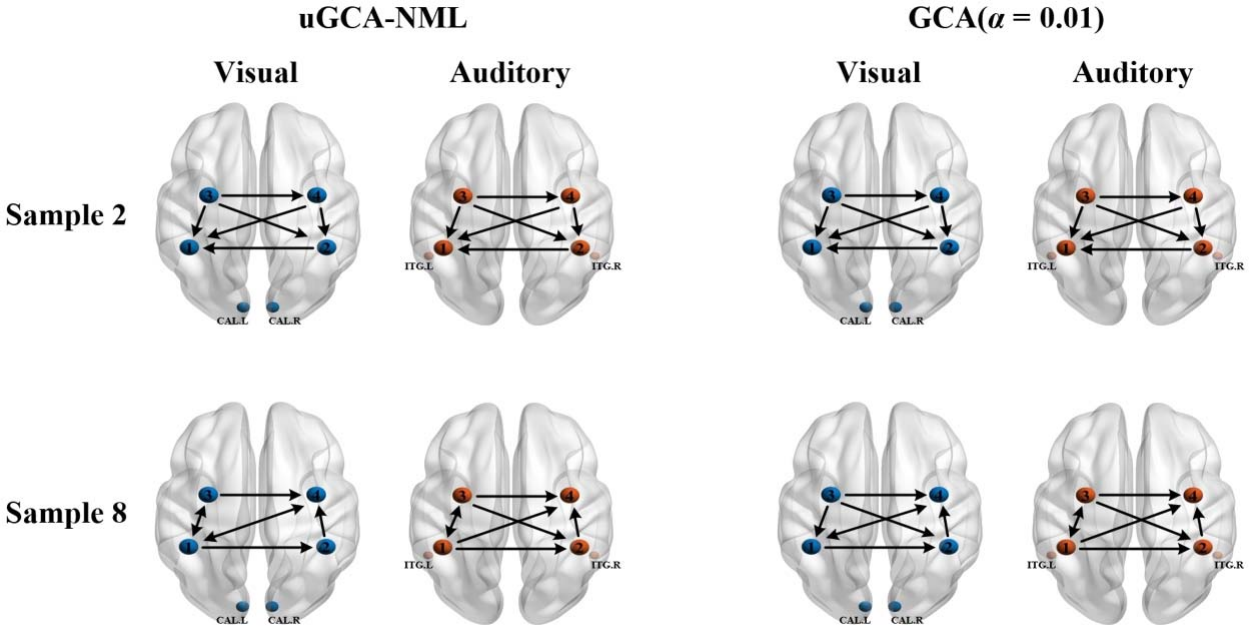
Mental arithmetic networks under different stimuli obtained by nonlinear uGCA and nonlinear conventional GCA.

As shown in Figure 6, in subject 2, their obtained mental arithmetic networks under different stimuli were completely consistent. In subject 8, only one connection edge in mental arithmetic network by nonlinear conventional GCA was inconsistent, and two edges were different for nonlinear uGCA-NML. Thus, using nonlinear modeling procedure could identify a high similarity of mental arithmetic networks under different stimuli.

Then, the network similarity between using nonlinear modeling and linear modeling was given by

$$S=\frac{\sum \sum \left(A\cap B\right)}{\sum \sum \left(A\cup B\right)}.$$ (19)

**Figure 7.**
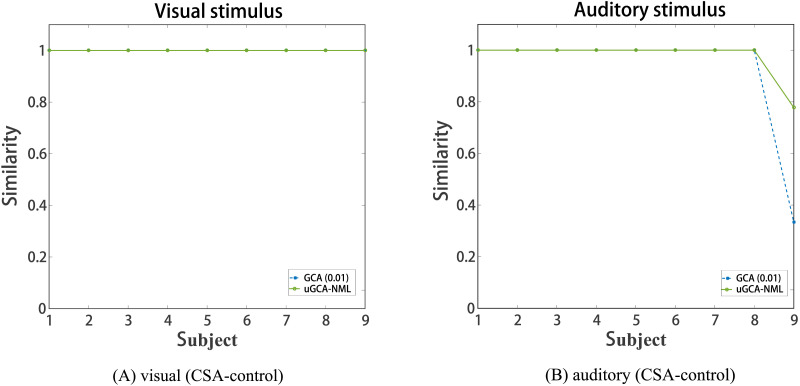
The obtained network similarity under the same stimulus between using nonlinear modeling and linear modeling.

Where *S* represents the measured similarity, *A* and *B* are the connection matrices of mental arithmetic networks under the same stimulus by nonlinear modeling and linear modeling, respectively. For the fMRI data under the same stimulus, nonlinear and linear modeling procedures were used to identify the causal connection, respectively, and then the similarities between them were compared. As shown in Figure 7, for both of methods, the most of causal connections between nonlinear modeling and linear modeling were exactly the same, except for the auditory stimulus fMRI data in subject 9.

As a result, due to some background noise terms, nonlinear characteristics in this mental arithmetic fMRI data seemed to be drowned out. Thus, for fMRI data, causal connections in the brain network can be well analyzed even using a linear procedure. In essence, linear AR model is the first-order approximation of a nonlinear system. For datasets with a high noise level, nonlinear characteristics will be drowned, and maybe only the first-order linear characteristics preserved. Furthermore, for the brain networks obtained by fMRI data, it will also retain linear causal connections. Using a nonlinear modeling procedure may result in high algorithm complexity, thus a simple linear modeling procedure is enough in most cases. Of course, it is necessary to use nonlinear modeling procedures to further analyze the deep characteristics of functional brain networks, such as analyzing and comparing topological properties of functional brain networks between normal people and patients.

#### Autism.

Autism spectrum disorder (ASD) refers to a group of complex neurodevelopmental disorders characterized by impairments in social interaction, verbal and nonverbal communication, and the presence of limited interests and repetitive behaviors (Lord et al., 2020; Lord, Elsabbagh, Baird, & Veenstra-Vanderweele, 2018; Sicile-Kira & Grandin, 2014). It is well known that diagnosing ASD is difficult because there is no specific medical diagnostic test, and the usual diagnosis is based on a doctor’s clinical observation and experience. Recently, many achievements have been made in revealing functional and anatomical abnormalities in the brain of ASD patients (Grove et al., 2019; Lord et al., 2020; Sicile-Kira & Grandin, 2014).

The imaging data used involved the resting-state fMRI data of ASD patients and normal people (NP). The ASD data contained 84 samples (42 female and 42 male) and were all from a data collection project: ABIDE I (https://ida.loni.usc.edu/) on the IDA website. For normal control group, we downloaded the datasets from NITRC website (https://www.nitrc.org/) which provided by V. J. Kiviniemi from the University of Oulu. The dataset included 66 female and 37 male (ages 20–23). Data preprocessing was performed on SPM12. According to previous studies (Alcalá-López et al., 2018), 36 regions of interest associated with ASD symptoms were selected. It was called a social brain network, containing four subsystems, limbic system, high-level cognitive system, visual-sensory system, and intermediate system.

**Figure 8.**
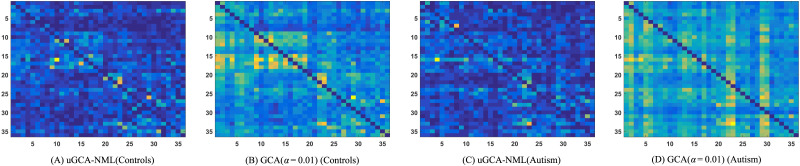
Causal connection matrices of social brain network identified by nonlinear uGCA and nonlinear conventional GCA.

Firstly, their obtained causal connections in social brain networks were shown in Figure 8. Obviously, there were some intuitive differences between the causal networks of ASDs and NP obtained using these two nonlinear modeling methods. Specifically, these obtained causal connections appeared to be haphazard in ASDs. In contrast, the obtained social brain networks of NP possessed more regularity and had different functional clusters among some certain nodes. For example, for the high-level cognitive system, there was a clear internal cluster of causal connections. But one of the differences was that nonlinear uGCA-NML identified relatively sparse networks.

**Figure 9.**
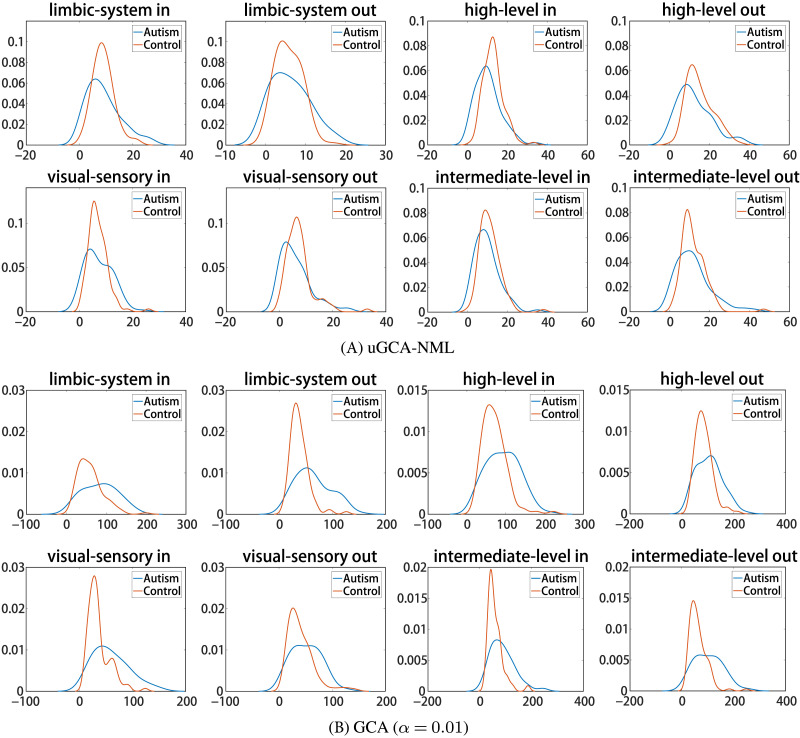
The out-degree and in-degree of subnetwork in social brain network within nonlinear uGCA and nonlinear conventional GCA. The four subnetworks are limbic, high-level, visual-sensory, and intermediate network.

To further reveal differences in obtained causal networks between ASDs and NP, out-degree and in-degree of four subnetworks in the social brain identified by these two methods were shown in Figure 9. Here, the in-degree represents the sum of (input) causal connections from other nodes to its own node, and the out-degree represents the sum of (output) causal connections from its own node to other nodes. For these subnetworks, whether using nonlinear uGCA-NML or nonlinear conventional GCA, the distributions of in-degree and out-degree of NP were intuitively more concentrated than that of ASDs. These suggested weaker group consistency for ASDs and broad overall distribution of social brain network characteristics. Thus, these results further implied the complexity of ASD symptoms.

On the other hand, the results showed that there were intuitive differences in social brain network between ASDs and NP, regardless of using nonlinear uGCA-NML or nonlinear conventional GCA. Specifically, for the limbic system and visual-sensory systems, nonlinear uGCA-NML revealed some differences in the causal networks between ASD and NP, implying that nonlinear uGCA considered ASD to be inconsistent in external information reception compared to NP. But nonlinear uGCA found little difference in out-degree between ASDs and NP for high-level cognitive system, and little difference in in-degree for intermediate system, these implying that nonlinear uGCA-NML believed the *output* of high-level cognitive and the *input* of intermediate systems in ASDs was no different from that of NP. In combination with Figure 8, internal connections of high-level cognitive network were sparser in ASD individuals than in normal individuals, from which it can be inferred that there may be some disorder in causal connections involving the high-level cognitive subnetwork in ASDs, and the same is true for the intermediate subnetwork. In general, to some extent, the differences in out-degree and in-degree between ASDs identified by nonlinear conventional GCA and normal individuals appear to be more pronounced.

**Figure 10.**
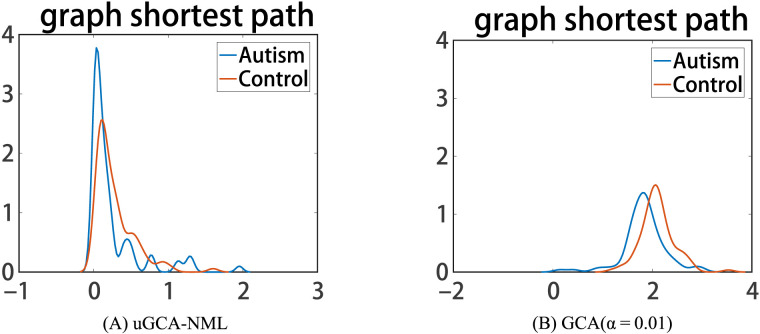
The small-world property of social brain networks obtained by nonlinear uGCA and nonlinear conventional GCA.

Next, the graph shortest paths of their social brain networks were further compared, as shown in Figure 10. Among them, nonlinear uGCA-NML showed that the distribution of graph shortest paths in social brain network of ASDs was more concentrated than that of NP. This implied that the ASD group had a higher consistency in the functional characteristics of their social brain networks when faced with social tasks (i.e., all individuals exhibited symptoms such as social difficulties). In contrast, nonlinear conventional GCA found some deviation in the center of the graphs shortest path distribution between ASDs and NP.

This result suggested that social brain network nodes in ASDs had shorter connected pathways (i.e., respond quickly to social tasks) than normal population when faced with social tasks, which was distinctly inconsistent with their clinical symptoms. Also, this could explain the greater difference in network out-degree and in-degree between ASD and normal individuals identified by nonlinear conventional GCA to some extent, which may actually be misjudged.

## DISCUSSIONS

As mentioned above, conventional GCA is still in a two-stage scheme, specifying model order by BIC/AIC and then determining causal effect by *F* test, which causes the inconsistency in selection benchmarks due to different mathematical theories, subjective selection of confidence level due to *F* test, and the extra algorithm complexity bringing by nested model (Li et al., 2020). Thus, we proposed a unified model selection approach for GCA based on MDL principle, named uGCA. Meanwhile, we all know that the nonlinear nature in functional brain networks is self-evident, but most conventional GCA methods identify causal effects through linear modeling. Therefore, we further incorporated Taylor’s approximate expansion technique into the proposed uGCA to identify causal connections in functional brain networks by nonlinear modeling procedure. It should be noted that the nonlinear uGCA and conventional nonlinear GCA actually used the same nonlinear modeling procedure. And instead of using BIC/AIC to determine the order of these nonlinear characteristics in conventional GCA, the order of high-order terms and cross-terms in uGCA method is determined by the description length, which is consistent with its whole modeling procedure. Thus in this sense, the superiority of nonlinear uGCA compared with nonlinear conventional GCA seems to outweigh its theoretical advantage in linear representations, in which its generalized model selection process is more consistent and simpler.

Through three-node synthetic network (including high-order term and cross-term, respectively), we revealed a noteworthy phenomenon that increasing noise levels in nonlinear systems will drown out some nonlinear characteristics. That is, with the increase of noise level, the nonlinear characteristics of high-order terms and cross-terms will be drowned out successively, and even affect the stability of its corresponding linear causal coupling at the same time. On the other hand, different from nonlinear conventional GCA, nonlinear uGCA-NML still can guarantee a relatively superior performance of causal connection identification. But nonlinear procedure seemed to be worse than linear procedure in the identification of some connections, as shown in Figure 1. We considered that nonlinear modeling involved incorporating higher order characteristics of the data into the model estimation process. And nonlinear characteristics in the data fitting might be more affected by the noise terms, which will generate additional uncertainties. As for linear modeling procedure, it only involved the description of first-order linear characteristics. Then, through a six-node synthetic network, it was further revealed that nonlinear uGCA were obviously prior to linear uGCA, which confirmed the necessity of using a nonlinear procedure to some extent.

Then, in the real fMRI data involving mental arithmetic tasks under different stimuli, the results showed that causal connections obtained using nonlinear modeling procedure and linear modeling procedure are largely consistent, which further revealed that nonlinear characteristics (high-order terms and cross-terms) in fMRI data may have been drowned out. In other words, applying a linear modeling procedure to investigate causal effect for real fMRI data (usually with a high noise level) may be appropriate enough. By the way, the advantages of linear uGCA over linear conventional GCA have been previously demonstrated in real fMRI experiment (Hu et al., 2021; Li et al., 2020). Then refer to the similarity measurement in Figure 7; the obtained mental networks using nonlinear modeling and linear modeling were highly similar, whether the uGCA or conventional GCA, so the advantage of nonlinear uGCA over nonlinear conventional GCA was self-evident. Next, for resting-state fMRI data involving ASDs and NP, uGCA-NML method revealed that ASDs showed a more consistent and concentrated distribution for graph shortest paths of social brain network, as shown in Figure 10. But for the four subnetworks, the consistency of its out-degree and in-degree distribution was obviously worse than in NP, as shown in Figure 9. Therefore, it can be concluded that ASDs may have some common dysfunction symptoms in the integration of social functions, and the individual differences of ASDs in four social brain subnetworks may be diverse. These are all consistent with ASD-related symptoms and the complex and extensive pathology may lead to significant differences in the inner connectivity of these four social brain subnetworks in ASDs. In this sense, nonlinear uGCA-NML may have certain advantages in distinguishing ASDs from NP.

## CONCLUSION

In this research, we incorporated the unified model selection framework with a nonlinear modeling procedure, which distilled the idea of Taylor’s approximate expansion, where a nonlinear uGCA approach was proposed. Through synthetic data and fMRI data experiments, the proposed uGCA methods showed a superior identification performance in nonlinear characteristics compared with conventional two-stage GCA, which will be more available in further functional brain network investigation. Meanwhile, we found that the nonlinear characteristics of high-order terms and cross-terms will be successively drowned out by the increasing noise level. Especially for the real fMRI data, we suggest that a simple linear modeling procedure for causal investigation may be appropriate enough.

On the other hand, uGCA methods can deal with different kinds of the noise terms automatically due to its model selection strategy involving modeling the noise term. And adopting a minimax solution for the inherent redundancy in earlier coding scheme, uGCA-NML method ensures a sharper description length with stochastic complexity and universal process, which actually applies a normalized maximum likelihood coding form for the generalized model selection issues. In this sense, uGCA-NML method can provide a more generic scheme for nonlinear modeling of causal connections. On the contrary, conventional GCA need additional techniques to accommodate different noise sources, which further bring in the inconsistency of mathematical theories and the subjectivity in model selection. Specifically, these will lead to more false positives and false negatives in the causal identification.

## AUTHOR CONTRIBUTIONS

Fei Li: Conceptualization; Data curation; Formal analysis; Investigation; Methodology; Resources; Software; Validation; Visualization; Writing – original draft; writing – review & editing. Qiang Lin: Funding acquisition; Supervision. Xiaohu Zhao: Funding acquisition; Project administration; Resources. Zhenghui Hu: Conceptualization; Funding acquisition; Project administration; Writing – review & editing.

## FUNDING INFORMATION

Zhenghui Hu, National Key Research and Development Program of China, Award ID: 2018YFA0701400. Zhenghui Hu, Public Projects of Science Technology Department of Zhejiang Province, Award ID: LGF20H180015. Xiaohu Zhao, Science and Technology Commission of Shanghai Municipality, Award ID: 201409002200.

## TECHNICAL TERMS

Granger causality analysis: Determines whether the historical information of exogenous variables is helpful in predicting the information of endogenous variables.
Model selection: Using a model selection strategy to select an optimal descriptive model among the candidate models.
F-test: A statistical inference that compares the variances of two datasets to determine whether there is a significant difference between them.
Unified Granger causality analysis: The proposed method based on the minimum description principle, a unified description length guided Granger causality analysis.
Minimum description length principle: A general model selection strategy, rooted in coding theory and stochastic complexity.
Ground truth: Truth value, true valid value, or standard answer, physically true existence.
Taylor’s expansion: An approximate expression using polynomials to approach the real physical model.
TPR: True positive rate, the number of obtained true connections divided by the number of all true connection in real existed system.
TNR: True negative rate, the percentage of non-existent connections that are correctly judged to be non-existent.
